# Supplementary material for: MetaRibo-Seq measures translation in microbiomes
Source: Nat Commun. 2020 Jun 29;11:3268. doi: 10.1038/s41467-020-17081-z (PMC7324362; doi:10.1038/s41467-020-17081-z)
Supplement: Supplementary file 10 — Supplementary Data 7 [file 41467_2020_17081_MOESM10_ESM.zip › File2/Confidence_High_Taxonomy/29810_out.krona.html]

Javascript must be enabled to view this page.

members
magnitude
magnitudeUnassigned
count
unassigned
taxon
rank

29810\_out

138

superkingdom
138
2

138
1239
phylum

138
186801
class

order
186802
138

186803
137
family

136
39491

SRS011084\_contig\_number\_38102SRS012849\_contig\_number\_24616SRS013940\_contig\_number\_21845SRS013951\_contig\_number\_39869SRS013965\_contig\_number\_9973SRS014313\_contig\_number\_4382SRS014412\_contig\_number\_18572SRS014683\_contig\_number\_contig-100\_4.125049SRS014948\_contig\_number\_19539SRS015217\_contig\_number\_18932SRS015578\_contig\_number\_3371SRS015794\_contig\_number\_contig-100\_15.94098SRS016018\_contig\_number\_12491SRS016056\_contig\_number\_contig-100\_195.195SRS016203\_contig\_number\_2794SRS016495\_contig\_number\_19505SRS017103\_contig\_number\_43625SRS017191\_contig\_number\_contig-100\_21.124338SRS017433\_contig\_number\_11003SRS018427\_contig\_number\_15735SRS018623\_contig\_number\_21094SRS019030\_contig\_number\_7768SRS019068\_contig\_number\_118111SRS019267\_contig\_number\_8644SRS019397\_contig\_number\_11911SRS019601\_contig\_number\_4601SRS020869\_contig\_number\_9007SRS021484\_contig\_number\_29015SRS022137\_contig\_number\_17809SRS022609\_contig\_number\_17234SRS023346\_contig\_number\_contig-100\_86.56070SRS024075\_contig\_number\_3981SRS024492\_contig\_number\_13605SRS042284\_contig\_number\_851SRS042628\_contig\_number\_35163SRS043001\_contig\_number\_6581SRS043411\_contig\_number\_18141SRS043667\_contig\_number\_5854SRS043701\_contig\_number\_17511SRS043768\_contig\_number\_7404SRS044535\_contig\_number\_24885SRS045004\_contig\_number\_contig-100\_317.187094SRS046717\_contig\_number\_4525SRS047433\_contig\_number\_19607SRS048060\_contig\_number\_13548SRS048870\_contig\_number\_23197SRS049446\_contig\_number\_15545SRS049959\_contig\_number\_45209SRS050422\_contig\_number\_14477SRS050925\_contig\_number\_36408SRS050941\_contig\_number\_3215SRS050998\_contig\_number\_16767SRS052027\_contig\_number\_contig-100\_191.191SRS052697\_contig\_number\_44039SRS053214\_contig\_number\_19400SRS053335\_contig\_number\_14232SRS053356\_contig\_number\_37122SRS053398\_contig\_number\_21075SRS055966\_contig\_number\_contig-100\_619.620SRS055982\_contig\_number\_18346SRS056519\_contig\_number\_6844SRS057478\_contig\_number\_contig-100\_118.63225SRS058723\_contig\_number\_5598SRS063190\_contig\_number\_10694SRS063370\_contig\_number\_11218SRS064973\_contig\_number\_contig-100\_1139.1139SRS075021\_contig\_number\_1569SRS075773\_contig\_number\_31681SRS075821\_contig\_number\_25001SRS076804\_contig\_number\_16628SRS077024\_contig\_number\_contig-100\_17402.63111SRS077194\_contig\_number\_contig-100\_53.192073SRS077231\_contig\_number\_15872SRS077294\_contig\_number\_12519SRS077335\_contig\_number\_contig-100\_160.59865SRS077502\_contig\_number\_16646SRS078176\_contig\_number\_21684SRS098073\_contig\_number\_15869SRS098571\_contig\_number\_contig-100\_129.368659SRS098644\_contig\_number\_45373SRS098717\_contig\_number\_23270SRS101376\_contig\_number\_28369SRS103987\_contig\_number\_36690SRS1041036\_contig\_number\_4845SRS1041037\_contig\_number\_7177SRS1041090\_contig\_number\_5601SRS1041091\_contig\_number\_17631SRS1041129\_contig\_number\_3298SRS1041133\_contig\_number\_24496SRS1041139\_contig\_number\_846SRS1041144\_contig\_number\_2740SRS104197\_contig\_number\_contig-100\_254.3304SRS104311\_contig\_number\_3925SRS104327\_contig\_number\_5037SRS104400\_contig\_number\_55908SRS104975\_contig\_number\_11797SRS105153\_contig\_number\_contig-100\_9.229404SRS1055049\_contig\_number\_13498SRS1055067\_contig\_number\_2580SRS142503\_contig\_number\_46615SRS142599\_contig\_number\_contig-100\_385.175356SRS142890\_contig\_number\_45788SRS143070\_contig\_number\_contig-100\_23.116598SRS143342\_contig\_number\_15395SRS143598\_contig\_number\_17092SRS143991\_contig\_number\_contig-100\_7896.7896SRS144135\_contig\_number\_33279SRS144362\_contig\_number\_45243SRS144506\_contig\_number\_56618SRS144603\_contig\_number\_1043SRS144714\_contig\_number\_12585SRS146764\_contig\_number\_4899SRS146812\_contig\_number\_contig-100\_85.321477SRS147139\_contig\_number\_35832SRS147271\_contig\_number\_39980SRS147346\_contig\_number\_46904SRS147614\_contig\_number\_9387SRS147766\_contig\_number\_15576SRS147977\_contig\_number\_contig-100\_57.87426SRS148196\_contig\_number\_39931SRS148253\_contig\_number\_14131SRS148424\_contig\_number\_30887SRS148721\_contig\_number\_53903SRS148817\_contig\_number\_1654SRS149244\_contig\_number\_9073SRS149325\_contig\_number\_15800SRS149784\_contig\_number\_contig-100\_25.166627SRS893252\_contig\_number\_16589SRS893259\_contig\_number\_contig-100\_159.53990SRS893279\_contig\_number\_4860SRS893295\_contig\_number\_11047SRS893341\_contig\_number\_4868SRS893369\_contig\_number\_contig-100\_234.72066SRS893373\_contig\_number\_20401SRS893378\_contig\_number\_21328SRS971276\_contig\_number\_25236
species

genus
841
1


SRS077641\_contig\_number\_17986
species
1
166486

1
541000
family

1
1263
genus

41978
1
species

SRS014235\_contig\_number\_27435
